# Fluctuating asymmetry based on fractal geometry does not track environmental stress in an intertidal habitat‐forming fucoid

**DOI:** 10.1111/jpy.70221

**Published:** 2026-08-12

**Authors:** Antonio Flores‐Moya, Ignacio J. Melero‐Jiménez, María J. García‐Sánchez, Elena Bañares‐España

**Affiliations:** ^1^ Departamento de Botánica y Fisiología Vegetal Universidad de Málaga Málaga Spain

**Keywords:** environmental stress, fluctuating asymmetry, fractal, *Fucus limitaneus*, self‐symmetry

## Abstract

The availability of easy‐to‐measure and inexpensive biomarkers is a challenge for monitoring environmental stress in organisms. Although it has been proposed that non‐directional deviations from a symmetrical pattern in morphological traits (called fluctuating asymmetry. FA) may be directly related to environmental stress, this postulate has been questioned. Self‐similarity symmetry (fractal) is one possible approach to addressing FA analysis, but there are very few studies with this approach, and the results have been inconclusive. Here, we evaluate whether fractal descriptors of thallus morphology in the habitat‐forming fucoid *Fucus limitaneus* reflect environmental stress across multiple contexts, including seasonal variation, vertical gradients in the intertidal zone, and the possible long‐term impact of an invasive species. Our results show that fractal metrics did not vary consistently across these contrasting environmental conditions, suggesting that in this system, FA based on fractal geometry does not reliably track environmental stress. This is possibly because stress is expressed through alternative ecological and biomechanical processes rather than through modifications of thallus self‐similarity.

**Abbreviations**
ANOVA: analysis of variance
*D*
: fractal dimension
FA: fluctuating asymmetry
*S*_yx_
: standard error of the estimate

## INTRODUCTION

Identifying easily measurable and inexpensive biomarkers of environmental stress remains a major challenge for environmental management and monitoring. Non‐directional deviations from a perfect symmetrical pattern in morphological traits, called fluctuating asymmetry (FA), constitute one of the bases of the eco‐evolutionary theory of developmental instability, which postulates that FA arises when an individual, throughout its ontogeny, diverts resources away from the developmental mechanisms that ensure symmetry to compensate for environmental or genetic stress (Møller & Swaddle, 1997). For this reason, it was proposed that FA measures could be used as estimators of environmental stress, although this assumption has been questioned (Gavrikov et al., 2023; Májeková et al., 2024; Palmer, 1999; Zverev & Kozlov, 2021). Most FA studies have focused on bilateral symmetry, but other types of symmetry, such as self‐similarity or fractal symmetry (Mandelbrot, 1977, 1982), may also provide insights into developmental instability. Fractal structures can arise through different forms of self‐similarity, including branching (hierarchical) fractals, boundary fractals defined by irregular perimeters, and statistical self‐similarity, in which scaling relationships hold across ranges rather than exact repetitions. In this context, fractal dimension (*D*), which quantifies the degree of space‐filling of a structure, has been proposed as an indicator of stress in organisms exhibiting self‐similar patterns (Graham et al., 1993). However, the response of *D* to stress is not necessarily unidirectional and may depend on the balance between growth, damage, and structural reorganization processes within each system. Indeed, previous studies in different levels of organization ranging from tissues to the full body of organisms have shown that *D* may decrease (Alados et al., 1998; Escós et al., 2000; Goldberger et al., 2002), remain stable (Schandorff, 1997), or even increase (Tracy et al., 1995) under environmental stress (reviewed by Graham et al., 2010). When *D* is estimated using the box‐counting method, the standard error of the estimate (*S*_yx_), representing the deviation from the ideal fractal scaling, has been suggested as an alternative proxy of FA. Indeed, Tracy et al. (1995) found that *S*_yx_ computed for *Fucus* increased under stress conditions. However, given that there are hardly any FA studies based on self‐symmetry in which *S*_yx_ has been reported, it has been suggested that its ecological relevance remains unclear (Graham et al., 2010).

In marine macroalgae, morphology is shaped not only by developmental processes but also by strong external mechanical forces, including wave action, drag, and breakage (Hayes et al., 2024; Starko et al., 2015). These forces can modify thallus architecture through pruning and selective survival, potentially decoupling developmental instability from observable morphological traits. Therefore, the applicability of FA metrics in these organisms may depend on how environmental stress interacts with both growth and mechanical constraints.

The habitat‐forming brown seaweed *Fucus* provides a suitable model to explore these questions. Its flattened thallus grows through dichotomous branching driven by apical cells, producing a hierarchical and self‐similar structure (Corbit & Garbary, 1995). In addition to this branching architecture, *Fucus* exhibits boundary fractal properties due to the irregularity of its thallus perimeter. Moreover, branching patterns may be modified by environmental factors, such as grazing or mechanical disturbance, through the formation of adventitious branches (Kinnby et al., 2019), further influencing structural complexity. From a theoretical perspective, branching represents an efficient strategy for space‐filling, and changes in branching frequency are expected to influence fractal dimension.

Previous studies have attempted to use deviations from ideal fractal patterns as indicators of environmental stress in *Fucus*. Although early work suggested a potential relationship (Tracy et al., 1995), subsequent studies reported inconclusive results when comparing populations under contrasting stress conditions (Melero‐Jiménez et al., 2017). Similarly, analyses based on bilateral traits have revealed variability in FA without clear links to individual fitness (Stige et al., 2007), highlighting the need for further evaluation of FA metrics in this system.

Here, we tested whether the FA, based on self‐symmetry of the thallus of *Fucus limitaneus*, can be related to environmental stress. For this purpose, we used two sets of observational data recorded in populations from the Strait of Gibraltar (Figure 1a). The first one was based on the comparison of deviations of the fractal pattern of *F. limitaneus* between seasons, coinciding with the coldest and warmest months (the former, when biomass was lowest and the latter corresponding to the reproductive peak and highest biomass; Sánchez de Pedro et al., 2023), and at different heights within the intertidal zonation. Thalli of *F. limitaneus* located at the upper limit of the distribution show greater physiological stress and a lower growth rate than those in the middle zone (Martins et al., 2019; Melero‐Jiménez et al., 2017). For the second dataset, we monitored two populations on the northern side of the Strait of Gibraltar in a long‐time series (2011–2024) during which a drastic decline in populations was recorded between 2014 and 2018. (Since then, they have not recovered.) The results from both data sets indicate that deviations from an ideal fractal pattern in *F. limitaneus* (as evaluated by *D* and S_yx_) remained largely unchanged across populations exposed to contrasting levels of stress and, notably, throughout a time series during which a marked population decline occurred.

**FIGURE 1:**
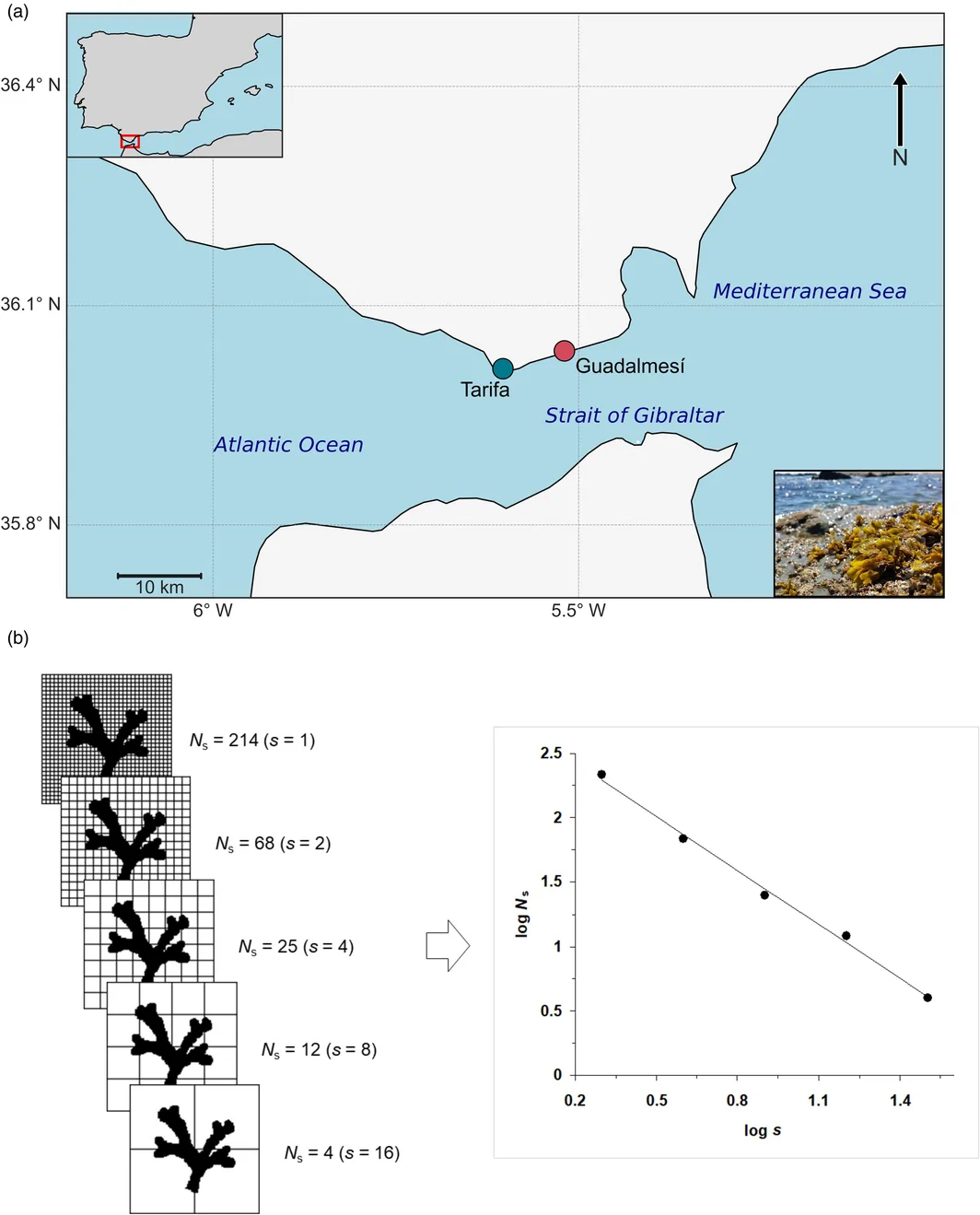
Map of the sampling sites of populations of *Fucus limitaneus* in the Strait of Gibraltar (Guadalmesí and Tarifa) and thalli growing in the intertidal from Guadalmesí (a), and schematic diagram of the box‐counting procedure (b; modified from Tracy et al., 1995) to compute the fractal dimension (*D*, absolute value of the linear fit) and the standard error of the estimate (*S*_yx_, square root of the residual mean square of the ANOVA of the linear fit). Photograph author: Dr. R. Sánchez de Pedro (freely accessible image of through Wikimedia Commons: https://commons.wikimedia.org/wiki/File:Fucus_guiryi_Strait_of_Gibraltar_SouthSpain.jpg).

## MATERIALS AND METHODS

### Sampling sites and collections

*Fucus limitaneus* is the main habitat‐forming seaweed in the intertidal of the Strait of Gibraltar (Flores‐Moya et al., 2021; Sánchez de Pedro et al., 2023). To study the effect of seasonality and height in the vertical distribution on self‐similarity of *F. limitaneus*, samples were collected at Tarifa, northern Strait of Gibraltar (Figure 1a), in February and August 2022, along the mid‐range and upper limit of the alga in the intertidal, as described in Melero‐Jiménez et al. (2017). Long‐term sampling (in years 2011, 2014, 2018, 2021, and 2024, in midsummer) was performed in two populations of *F. limitaneus* from the northern Strait of Gibraltar (Guadalmesí and Tarifa; Figure 1a).

In all cases, samplings were carried out during low tide of a spring tide. In short, a 5‐m (length) transect was located parallel to the shoreline in the mid‐range of the vertical distribution of *Fucus limitaneus* and at the upper limit in the case of the study of the effect of seasonality and height on self‐similarity. A 15 × 15 cm quadrat was placed every 20 cm along the transect, and a single thallus was collected from the center of the quadrat. The sampling stopped when the 15th thallus (in the study of the effect of seasonality and height on the self‐similarity) or 21st thallus (in the long‐term time series) lacking grazing marks was collected; this precaution was taken because grazing marks alter the thallus shape and, consequently, the self‐symmetry. Thus, individuals showing no damage were selected (~15% of thalli showed herbivore marks, especially by the fish *Sarpa salpa*). The samples were transported to the laboratory in plastic bags in an ice box. In the laboratory, the thalli were rinsed with fresh water, and any visible epiphytes were removed. Individuals of *F. limitaneus* tend to form aggregates, so it was necessary to separate them from the basal zone, isolating individual thalli from the attachment disk.

In the long‐term study, population cover (expressed as percentage of substrate covered by *Fucus limitaneus*) was recorded in the first five 15 × 15 cm quadrats.

### Measurement of FA based on self‐similarity of the thallus of *Fucus limitaneus*

As a proxy of the FA in *Fucus limitaneus* individuals, we estimated the deviations of the self‐symmetry of the area of thalli by using the box‐counting procedure (Mandelbrot, 1982; Figure 1b). For this purpose, thalli were placed between two transparent acetate foils, avoiding overlapping the blades, and they were scanned, without changing the image scale, in TIFF format (300 ppi). The scanned images were superimposed on acetate foils with impressed grids of exponentially increasing box sizes (0.125, 0.25, 0.5, 1, 2, and 4 cm^2^). To minimize experimental error, all the stored scanned images were processed simultaneously in 2024, in a blind fashion (i.e., the person processing the sample did not know the identity of the sample being measured nor the hypothesis being tested) by an independent observer. In this way, the possibility of unintentional confirmation bias was avoided (Kozlov, 2025), minimizing the likelihood of Type I error. The number of boxes in which at least part of the thallus occurred were counted using the image analysis system Visilog 6.3 (Noesis, France). The absolute value of the slope of the linear regression line log of the number of occupied boxes against the log of the size of each box is *D* (Figure 1b). The value of *S*_yx_ was computed as the square root of the residual mean square of the analysis of variance (ANOVA) of the linear fit (Figure 1b).

Since positioning of the grids on the image of thalli could affect the results of a box count procedure (quantization error; Bouda et al., 2016), the process was repeated three times (i.e., technical replicates) by randomly repositioning every thallus over the grids. Since the coefficient of variation of the three technical replicates of *D* and *S*_yx_ per thallus never exceeded 0.6% of the mean for the 260 thalli analyzed, the overall mean of the three technical replicates was selected.

### Statistical analysis

Given the observational nature of the study, factors such as site, seasonality, and height in the intertidal zone were not fully orthogonal, and factorial designs may have led to pseudoreplication and pseudofactorial interpretations (Hurlbert, 1984, 2013). Accordingly, conservative analytical approaches were adopted. The effect of seasonality and height on fractal descriptors (*D* and *S*_yx_) was assessed using a one‐way ANOVA on the combined factor (seasonality × height), thereby avoiding factorial inference. For the long‐term dataset, the effect of year and population on cover was analyzed using a linear model including both factors as fixed terms (cover ~ year + population). In contrast, temporal variation in *D* and *S*_yx_ was evaluated using one‐way ANOVAs conducted separately within each population. Although some datasets showed deviations from homogeneity of variances, untransformed data were used given the robustness of ANOVA in balanced designs (Underwood, 1997). The significance level was set at 0.01 to reduce Type I errors, and Tukey's tests were applied when appropriate. Additionally, the effect size (η^2^ in the one‐way ANOVAs and η ^2^_p_ in the linear model), i.e., the proportion of variance in the dependent variable explained by a given factor, was computed by using the sum of squares. The benchmarks proposed by Cohen (1988) were used to classify the effect size: small, 0.01–0.06; moderate, 0.06–0.14; and large, >0.14. All statistical analyses and figures were generated using Python 3.11.9.

## RESULTS

### Effect of seasonality and height in the vertical range on the FA of *Fucus limitaneus*

Fractal descriptors (*D* and *S*_yx_) were compared between thalli collected in February and August at two heights within the vertical range in the intertidal, to assess potential differences in environmental conditions during ontogeny (Figure 2a,b). When analyzed using a one‐way ANOVA on the combined factor (seasonality × height), no significant differences in *D* were detected among the four combinations, with a moderate effect size (*F*_3,56_ = 1.36, *p* = 0.265, η^2^ = 0.07; Appendix S1: Table S1), with an overall mean value of 1.16 ± 0.11 (Figure 2a). Similarly, *S*_yx_ values did not differ among season–height combinations, with a negligible effect size (*F*_3,56_ = 0.13, *p* = 0.939, η^2^ < 0.01; Appendix S1: Table S1), yielding an overall mean of 0.06 ± 0.02 (Figure 2b). These results indicate no detectable effect of seasonality or position in the vertical range on fractal‐based FA of *Fucus limitaneus*, despite the expected differences in environmental conditions (e.g., desiccation and thermal stress).

**FIGURE 2:**
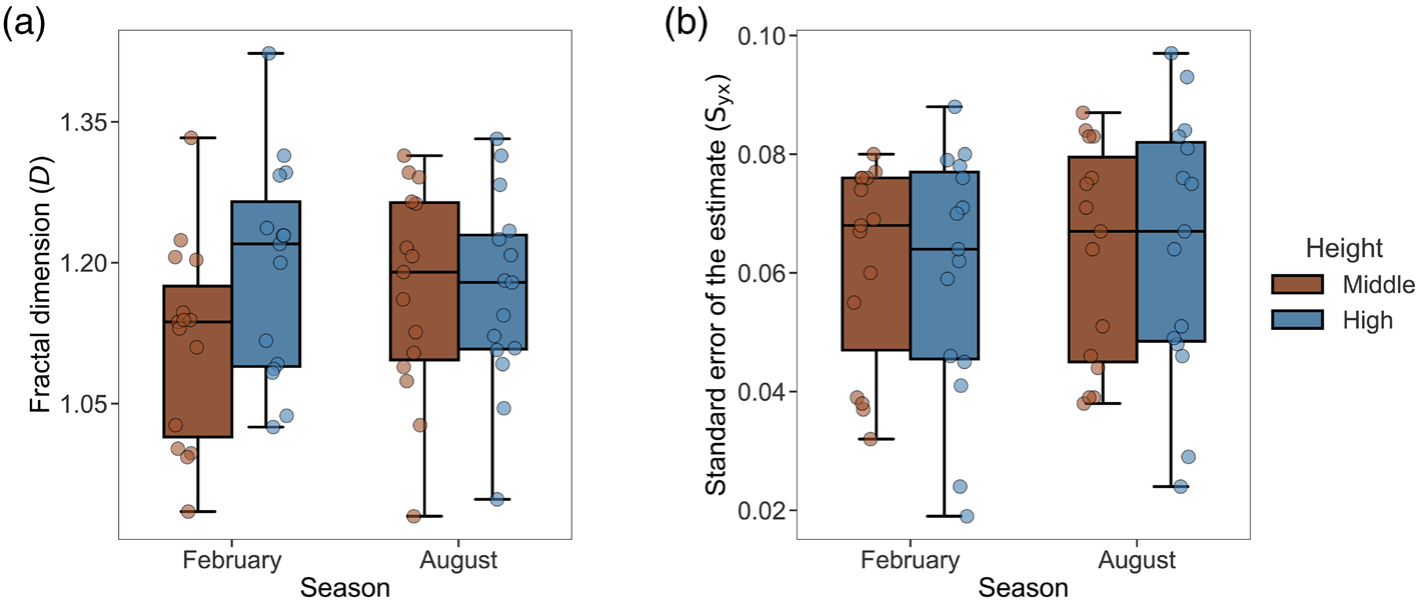
Box‐whisker plot (minimum, first quartile, median, third quartile and maximum; *n* = 15) for the fractal dimension *D* (a) and the standard error of the estimate *S*_yx_ (b), in *Fucus limitaneus* from Tarifa.

### Time series (2011–2024) of cover and FA of *Fucus limitaneus*

A long‐term survey of *Fucus limitaneus* populations from the Strait of Gibraltar was carried out. For this purpose, the substrate cover percentage was recorded in two populations along the time series 2011–2024 (Figure 3a). It is important to highlight that during this time period, there was a considerable disruption of the algal communities of the Strait of Gibraltar due to the arrival and spread of the invasive brown alga *Rugulopteryx okamurae* (García‐Gómez, Florido, Olaya‐Ponzone, Sempere‐Valverde, & Megina, 2021). Cover differed significantly among years and between populations, but the effect of year was substantially stronger than that of population. The partial effect size associated with year was more than four times greater than that of population (linear model including year and population as fixed terms: year, *F*_4,44_ = 76.65, *p* < 0.001, η^2^_*p*_ = 0.87; population, *F*_1,44_ = 11.26, *p* = 0.002, η^2^_*p*_ = 0.20; Appendix S1: Table S2). Mean cover was high in 2011 and 2014 (38.1% ± 10.1%) but declined sharply from 2018 onward, reaching 11.4% ± 6.9% during the period 2018–2024 in both populations (Figure 3a).

**FIGURE 3:**
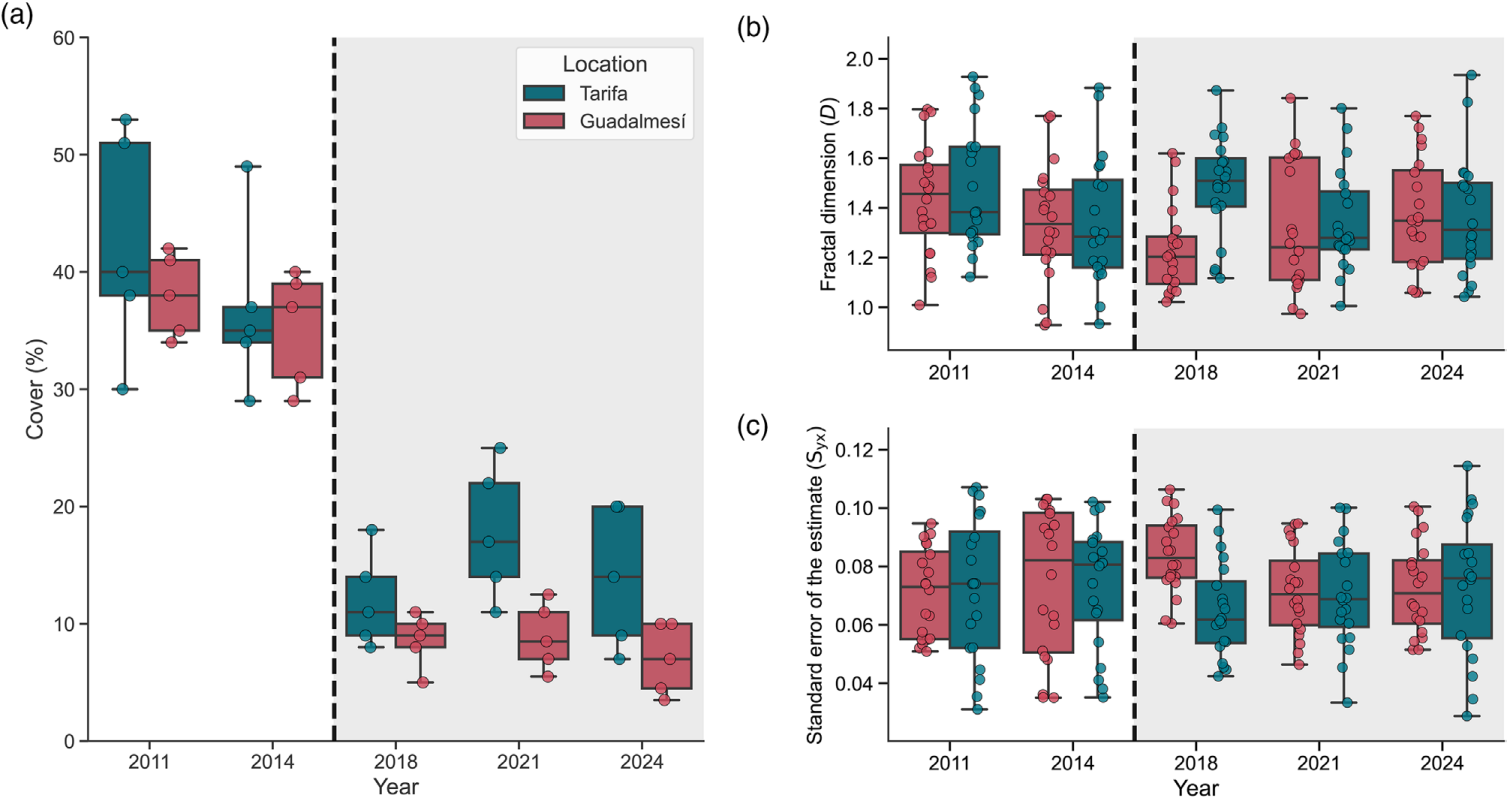
Box‐whisker plot (minimum, first quartile, median, third quartile and maximum) for cover (a; *n* = 5), the fractal dimension *D* (b; *n* = 20) and the standard error of the estimate *S*_yx_ (c; *n* = 20) of *Fucus limitaneus* in August 2011, 2014, 2018, 2021, and 2024, in two populations from the Strait of Gibraltar (Guadalmesí and Tarifa). The gray background indicates the period corresponds to the arrival and spreading of the invasive *Rugulopteryx okamurae* (from 2015 onward).

To evaluate whether this decline was reflected in thallus morphology, fractal descriptors (*D* and *S*_yx_) were analyzed across the same time series (Figure 3b,c). One‐way ANOVAs conducted separately within each population detected no significant temporal variation in *D*, with a moderate effect size in both locations (Guadalmesí: *F*_4,95_ = 2.23, *p* = 0.071, η^2^ = 0.09; Tarifa: *F*_4,95_ = 1.98, *p* = 0.103, η^2^ = 0.08; Appendix S1: Table S3), with an overall mean value of 1.37 ± 0.24 across populations and years (Figure 3b). Likewise, *S*_yx_ did not vary significantly across years within either population, with a small to moderate effect size (Guadalmesí: *F*_4,95_ = 1.85, *p* = 0.125, η^2^ = 0.07; Tarifa: *F*_4,95_ = 0.54, *p* = 0.704, η^2^ = 0.02; Appendix S1: Table S4), yielding an overall mean value of 0.07 ± 0.02 (Figure 3c). Descriptive statistics (mean ± *SD*) for both fractal descriptors across populations and years are provided in Appendix S1: Table S5.

Thus, despite a marked decline in population cover, fractal descriptors of thallus architecture remained comparatively stable, suggesting that environmental stress may primarily affect population abundance rather than the self‐similar structure of surviving individuals.

## DISCUSSION

The identification of reliable and inexpensive biomarkers remains a central challenge in the monitoring of natural populations exposed to environmental stress. In this study, we evaluated whether fractal‐based estimators of FA in *Fucus limitaneus* respond consistently to environmental gradients across spatial, seasonal, and long‐term contexts. Despite the clear contrast among these scenarios, fractal descriptors (*D* and *S*_yx_) remained largely invariant.

In intertidal fucoids, thallus morphology derives from the interaction between developmental processes and strong external mechanical forces. Decades of work have demonstrated that wave‐induced drag, breakage, and hydrodynamic loading play a dominant role in shaping macroalgal form (Blanchette, 1997; Gaylord et al., 1994; Martone et al., 2012). Further recent studies show that hydrodynamic conditions influence branching patterns, growth, and phenotypic variation in fucoids, often through complex interactions with grazing and habitat structure (Coleman & Martone, 2024; Hayes et al., 2024). Under these conditions, morphology is not solely the outcome of growth patterns, but also of mechanical pruning and the selective survival of individuals capable of withstanding physical stress (Boller & Carrington, 2006; Martone & Denny, 2008). Furthermore, variation in seaweed form across environmental gradients does not necessarily reflect developmental plasticity but may instead arise from biomechanical filtering and differential survival (Coleman & Martone, 2024). This mechanistic framework challenges the assumption that subtle deviations from idealized branching symmetry should directly track environmental stress.

The environmental contexts considered here represent distinct and non‐equivalent stress mechanisms. Seasonal variation is primarily associated with physiological constraints, such as temperature and growth dynamics, whereas vertical gradients in the intertidal zone involve desiccation, thermal stress, and exposure to air. In contrast, the long‐term decline observed in *Fucus limitaneus* populations is most plausibly linked to biotic interactions, particularly the invasion of *Rugulopteryx okamurae*, which imposes stress through competition, overgrowth, physical burial, and allelopathic effects on single‐ and few‐celled stages of development of fucoids (Gálvez‐Pastor et al., 2025; García‐Gómez et al., 2020; García‐Gómez, Florido, Olaya‐Ponzone, Rey Díaz de Rada, et al., 2021; García‐Gómez, Florido, Olaya‐Ponzone, Sempere‐Valverde, & Megina, 2021; Román & Vázquez, 2025; Sánchez de Pedro et al., 2022). Importantly, hydrodynamic forces also vary along the vertical gradient and may interact with these stressors, further influencing thallus morphology (Gaylord et al., 1994; Martone et al., 2012). These processes are unlikely to produce uniform morphological responses or to be captured by a single FA metric.

Despite these contrasting environmental pressures, fractal descriptors remained stable, even during a period of marked population decline. This decoupling between population‐level changes and individual morphology suggests that environmental stress in *Fucus limitaneus* may operate primarily through demographic processes rather than through gradual modification of thallus architecture. In particular, stress may act through the selective removal of individuals via breakage, dislodgement, or competitive exclusion, while surviving thalli retain a relatively consistent structural organization. This interpretation is consistent with previous work showing that fucoid morphology is strongly constrained by biomechanical performance and that individuals exposed to high stress may not exhibit intermediate forms but rather be removed from the population (Blanchette, 1997; Boller & Carrington, 2006). Consequently, traits based on overall geometry, such as the descriptors of the fractal symmetry of the thallus of *F. limitaneus*, may remain stable even under substantial environmental change because they reflect the architecture of surviving individuals rather than the full spectrum of developmental responses.

Our results, therefore, do not support a general relationship between fractal‐based FA and environmental stress in *Fucus limitaneus*. However, rather than indicating the absence of stress effects, they suggest that the selected FA descriptors are not aligned with the dominant pathways through which stress acts in this system. Alternative traits more directly linked to mechanical performance or population dynamics (such as breakage rates, tissue strength, or survival probability) may provide more informative indicators of stress in intertidal macroalgae.

Finally, it is important to consider the limitations inherent to fractal metrics. Recent studies have emphasized methodological and interpretative challenges in estimating and applying fractal dimensions to biological branching systems, cautioning against their use as universal indicators of functional or ecological processes (Cheeseman & Vrscay, 2022). We emphasize that we carefully followed the recommendations proposed by Graham (2021), Zverev and Kozlov (2021) and Kozlov (2025) in FA studies; in short, all the samples were randomly collected and the measurements of FA were conducted blind, supporting our general conclusion that self‐similarity pattern changes in *Fucus limitaneus* do not appear to be a reliable indicator of environmental stress in this system. In this context, our results highlight the importance of integrating fractal approaches with a mechanistic understanding of organismal biology when evaluating their ecological relevance.

## AUTHOR CONTRIBUTIONS

**Antonio Flores‐Moya:** Conceptualization (lead); data curation (lead); formal analysis (lead); funding acquisition (equal); investigation (lead); writing – original draft (lead); writing – review and editing (equal). **Ignacio J. Melero‐Jiménez:** Formal analysis (equal); investigation (equal); writing – original draft (equal); writing – review and editing (equal). **María J. García‐Sánchez:** Funding acquisition (equal); writing – original draft (equal); writing – review and editing (equal). **Elena Bañares‐España:** Funding acquisition (equal); writing – original draft (equal); writing – review and editing (equal).

## FUNDING INFORMATION

This study was financially supported by resources from the project PCIN‐2016‐090 (Ministerio de Economía y Competitividad, Spain) and “Ayudas a la investigación” from Instituto de Estudios Ceutíes 2018″ with the project “Variabilidad funcional de las poblaciones de *Fucus* del litoral de Ceuta: respuestas al cambio climático.” The edition of the manuscript was partially supported by the project PID2023‐152625NB‐I00 (Ministerio de Ciencia, Innovación y Universidades, Spain). Funding for open access charge: Universidad de Málaga/CBUA.

## Supporting information


**Appendix S1.** Supplementary statistical analyses.
**Table S1.** One‐way ANOVA testing the effect of seasonality × height on fractal descriptors (*D* and Syx) in *Fucus limitaneus*.
**Table S2.** Analysis of variance of a linear model testing the effects of year and population on the cover of *Fucus limitaneus* (model: cover ~ year + population).
**Table S3.** One‐way ANOVAs testing temporal variation in fractal dimension (*D*) within each population of *Fucus limitaneus*.
**Table S4.** One‐way ANOVAs testing temporal variation in Syx within each population of *Fucus limitaneus*.
**Table S5.** Mean (±*SD*) values of fractal descriptors (*D* and Syx) by population and year of *Fucus limitaneus*.

**Data S1.** Fractal dimension (*D*) and standard error of the estimate (*S*_yx_) computed in the linear fit by the box‐counting procedure, in thalli of *Fucus limitaneus* collected at Tarifa in two seasons and two heights of the vertical range of distribution in 2022 (*n* = 15).

**Data S2.** Cover of *Fucus limitaneus* (expressed as percentage of substrate covered by the alga; *n* = 5 quadrats of 15 × 15 cm) at two populations at the Strait of Gibraltar (Guadalmesí and Tarifa) along the time‐series 2011–2024.

**Data S3.** Fractal dimension (*D*) and standard error of the estimate (*S*_yx_) computed in the linear fit by the box‐counting procedure, in thalli of *Fucus limitaneus* collected at two populations from the Strait of Gibraltar (Guadalmesí and Tarifa) along the time‐series 2011–2024 (*n* = 20).

## Data Availability

All raw data and Python code required to reproduce the analyses, figures, and tables are provided as Supporting Information.
