# Supplementary material for: Fluctuating asymmetry based on fractal geometry does not track environmental stress in an intertidal habitat‐forming fucoid
Source: J Phycol. 2026 Aug 12;62(5):1597–605. doi: 10.1111/jpy.70221 (PMC13659371; doi:10.1111/jpy.70221)
Supplement: Supplementary file 1 [file JPY-62-1597-s001.pdf]

SUPPORTING INFORMATION

Supporting information for: **Fluctuating asymmetry based on fractal geometry does not track environmental stress in an intertidal habitat-forming fucoid**

Antonio Flores-Moya*, Ignacio J. Melero-Jiménez, María J. García Sánchez, Elena Bañares-España

Departamento de Botánica y Fisiología Vegetal, Universidad de Málaga, Málaga, Spain

*Corresponding author: floresa@uma.es

Appendix S1. Supplementary statistical analyses

This appendix provides the statistical results supporting the analyses presented in the main text. Tables S1–S4 summarize the ANOVA and linear model results used to evaluate the effects of seasonality, height, year, and population on cover and fractal descriptors (D and Syx) in *Fucus limitaneus*, including the corresponding effect size estimates (η^2 and η^2p). Table S5 presents descriptive statistics (mean ± SD) for D and Syx across populations and sampling years.

Table S1. One-way ANOVA testing the effect of seasonality × height on fractal descriptors (*D* and S_{yx}) in *Fucus limitaneus*.

Variable	Source	df	SS	F	P	η^2
D	Season × Height	3	0.0486	1.357	0.265	0.07
	Residual	56	0.6685			
S_{yx}	Season × Height	3	0.00016	0.134	0.939	<0.01
	Residual	56	0.0220			

Table S2. Analysis of variance of a linear model testing the effects of year and population on the cover of *Fucus limitaneus* (model: cover ~ year + population).

Source	df	SS	F	P	η^2_p
Year	4	8711.78	76.65	<0.001	0.87
Population	1	320.05	11.26	0.002	0.20
Residual	44	1250.18			

Note: year and population were treated as fixed factors. Model fitted using ordinary least squares.

Table S3. One-way ANOVAs testing temporal variation in fractal dimension (*D*) within each population of *Fucus limitaneus*.

**Population**	**Source**	**df**	**SS**	**F**	**P**	η^2
Guadalmesí	Year	4	0.4582	2.234	0.071	0.09
	Residual	95	4.8721			
Tarifa	Year	4	0.4286	1.982	0.103	0.08
	Residual	95	5.1348			

Table S4. One-way ANOVAs testing temporal variation in S_{yx} within each population of *Fucus limitaneus*.

**Population**	**Source**	**df**	**SS**	**F**	**P**	η^2
Guadalmesí	Year	4	0.00225	1.851	0.125	0.07
	Residual	95	0.0289			
Tarifa	Year	4	0.00095	0.544	0.704	0.02
	Residual	95	0.0416			

Table S5. Mean (± SD) values of fractal descriptors (*D* and S_{yx}) by population and year of *Fucus limitaneus*.

**Population**	**Year**	***D* (mean ± SD)**	**S_{yx} (mean ± SD)**
Guadalmesí	2011	1.435 ± 0.225	0.071 ± 0.015
	2014	1.337 ± 0.238	0.075 ± 0.025
	2018	1.229 ± 0.173	0.084 ± 0.013
	2021	1.319 ± 0.262	0.071 ± 0.015
	2024	1.374 ± 0.225	0.073 ± 0.016
Tarifa	2011	1.483 ± 0.246	0.072 ± 0.024
	2014	1.344 ± 0.259	0.073 ± 0.021
	2018	1.486 ± 0.204	0.065 ± 0.016
	2021	1.345 ± 0.206	0.070 ± 0.018
	2024	1.366 ± 0.242	0.073 ± 0.024
