# Supplementary material for: Fluctuating asymmetry based on fractal geometry does not track environmental stress in an intertidal habitat‐forming fucoid
Source: J Phycol. 2026 Aug 12;62(5):1597–605. doi: 10.1111/jpy.70221 (PMC13659371; doi:10.1111/jpy.70221)
Supplement: Supplementary file 2 [file JPY-62-1597-s003.docx]

Raw Data

Raw data set 1. Fractal dimension (*D*) and standard error of the estimate (S_yx_) computed in the linear fit by the box-counting procedure, in thalli of *Fucus limitaneus* collected at Tarifa in two seasons and two heights of the vertical range of distribution in 2022 (*n* = 15).

Season	Height	*D*	S_yx_

February	Middle	1.137	0.067

February	Middle	1.206	0.055

February	Middle	1.110	0.069

February	Middle	1.027	0.039

February	Middle	1.002	0.037

February	Middle	1.203	0.060

February	Middle	1.147	0.032

February	Middle	0.997	0.077

February	Middle	1.224	0.076

February	Middle	1.13	0.076

February	Middle	0.993	0.080

February	Middle	0.935	0.038

February	Middle	1.139	0.076

February	Middle	1.333	0.068

February	Middle	1.139	0.074

February	High	1.037	0.059

February	High	1.296	0.046

February	High	1.200	0.071

February	High	1.117	0.041

February	High	1.092	0.019

February	High	1.293	0.062

February	High	1.237	0.024

February	High	1.087	0.079

February	High	1.314	0.078

February	High	1.220	0.088

February	High	1.083	0.080

February	High	1.025	0.045

February	High	1.229	0.064

February	High	1.423	0.070

February	High	1.229	0.076

August	Middle	1.296	0.064

August	Middle	1.314	0.051

August	Middle	1.263	0.076

August	Middle	1.291	0.046

August	Middle	1.265	0.044

August	Middle	1.126	0.067

August	Middle	1.089	0.039

August	Middle	1.161	0.084

August	Middle	1.027	0.083

August	Middle	0.930	0.083

August	Middle	1.074	0.087

August	Middle	1.104	0.038

August	Middle	1.190	0.039

August	Middle	1.216	0.075

August	Middle	1.207	0.071

August	High	1.314	0.064

August	High	1.332	0.051

August	High	1.181	0.076

August	High	1.109	0.046

August	High	1.283	0.024

August	High	1.144	0.067

August	High	1.107	0.029

August	High	1.179	0.084

August	High	1.045	0.083

August	High	0.948	0.093

August	High	1.092	0.097

August	High	1.122	0.048

August	High	1.208	0.049

August	High	1.234	0.075

August	High	1.225	0.081

Raw data set 2. Cover of *Fucus limitaneus* (expressed as percentage of substrate covered by the alga; *n* = 5 quadrats of 15×15 cm) at two populations at the Strait of Gibraltar (Guadalmesí and Tarifa) along the time-series 2011-2024.

Population	Year	Cover (%)

Guadalmesí	2011	38

Guadalmesí	2011	41

Guadalmesí	2011	34

Guadalmesí	2011	42

Guadalmesí	2011	35

Guadalmesí	2014	37

Guadalmesí	2014	39

Guadalmesí	2014	31

Guadalmesí	2014	40

Guadalmesí	2014	29

Guadalmesí	2018	8

Guadalmesí	2018	5

Guadalmesí	2018	9

Guadalmesí	2018	10

Guadalmesí	2018	11

Guadalmesí	2021	9

Guadalmesí	2021	11

Guadalmesí	2021	7

Guadalmesí	2021	13

Guadalmesí	2021	6

Guadalmesí	2024	7

Guadalmesí	2024	10

Guadalmesí	2024	4

Guadalmesí	2024	10

Guadalmesí	2024	5

Tarifa	2011	30

Tarifa	2011	51

Tarifa	2011	38

Tarifa	2011	53

Tarifa	2011	40

Tarifa	2014	34

Tarifa	2014	37

Tarifa	2014	29

Tarifa	2014	49

Tarifa	2014	35

Tarifa	2018	14

Tarifa	2018	11

Tarifa	2018	9

Tarifa	2018	8

Tarifa	2018	18

Tarifa	2021	17

Tarifa	2021	22

Tarifa	2021	14

Tarifa	2021	25

Tarifa	2021	11

Tarifa	2024	14

Tarifa	2024	20

Tarifa	2024	7

Tarifa	2024	20

Tarifa	2024	9

Raw data set 3. Fractal dimension (*D*) and standard error of the estimate (S_yx_) computed in the linear fit by the box-counting procedure, in thalli of *Fucus limitaneus* collected at two populations from the Strait of Gibraltar (Guadalmesí and Tarifa) along the time-series 2011-2024 (*n* = 20).

Year	Population	*D*	S_yx_

2011	Guadalmesí	1.796	0.052

2011	Guadalmesí	1.487	0.091

2011	Guadalmesí	1.542	0.084

2011	Guadalmesí	1.625	0.055

2011	Guadalmesí	1.216	0.074

2011	Guadalmesí	1.787	0.053

2011	Guadalmesí	1.478	0.055

2011	Guadalmesí	1.139	0.063

2011	Guadalmesí	1.435	0.081

2011	Guadalmesí	1.562	0.064

2011	Guadalmesí	1.385	0.088

2011	Guadalmesí	1.607	0.058

2011	Guadalmesí	1.354	0.051

2011	Guadalmesí	1.009	0.088

2011	Guadalmesí	1.326	0.078

2011	Guadalmesí	1.121	0.090

2011	Guadalmesí	1.502	0.072

2011	Guadalmesí	1.337	0.095

2011	Guadalmesí	1.217	0.074

2011	Guadalmesí	1.771	0.055

2014	Guadalmesí	1.218	0.048

2014	Guadalmesí	1.408	0.101

2014	Guadalmesí	1.762	0.036

2014	Guadalmesí	1.226	0.093

2014	Guadalmesí	1.299	0.060

2014	Guadalmesí	1.503	0.051

2014	Guadalmesí	1.392	0.103

2014	Guadalmesí	1.270	0.065

2014	Guadalmesí	0.928	0.103

2014	Guadalmesí	0.992	0.091

2014	Guadalmesí	1.463	0.094

2014	Guadalmesí	1.770	0.035

2014	Guadalmesí	1.597	0.077

2014	Guadalmesí	1.448	0.035

2014	Guadalmesí	1.139	0.063

2014	Guadalmesí	1.519	0.087

2014	Guadalmesí	1.305	0.099

2014	Guadalmesí	1.193	0.098

2014	Guadalmesí	0.938	0.101

2014	Guadalmesí	1.365	0.049

2018	Guadalmesí	1.205	0.096

2018	Guadalmesí	1.469	0.077

2018	Guadalmesí	1.048	0.102

2018	Guadalmesí	1.620	0.074

2018	Guadalmesí	1.054	0.102

2018	Guadalmesí	1.074	0.075

2018	Guadalmesí	1.101	0.094

2018	Guadalmesí	1.064	0.077

2018	Guadalmesí	1.211	0.095

2018	Guadalmesí	1.253	0.106

2018	Guadalmesí	1.178	0.081

2018	Guadalmesí	1.114	0.091

2018	Guadalmesí	1.276	0.086

2018	Guadalmesí	1.389	0.069

2018	Guadalmesí	1.585	0.061

2018	Guadalmesí	1.203	0.076

2018	Guadalmesí	1.310	0.080

2018	Guadalmesí	1.257	0.089

2018	Guadalmesí	1.022	0.085

2018	Guadalmesí	1.148	0.062

2021	Guadalmesí	1.257	0.089

2021	Guadalmesí	1.614	0.057

2021	Guadalmesí	1.108	0.092

2021	Guadalmesí	1.314	0.080

2021	Guadalmesí	0.974	0.095

2021	Guadalmesí	1.659	0.050

2021	Guadalmesí	1.611	0.076

2021	Guadalmesí	1.600	0.059

2021	Guadalmesí	1.079	0.075

2021	Guadalmesí	1.619	0.075

2021	Guadalmesí	1.117	0.067

2021	Guadalmesí	1.132	0.065

2021	Guadalmesí	1.111	0.069

2021	Guadalmesí	1.298	0.060

2021	Guadalmesí	0.995	0.091

2021	Guadalmesí	1.226	0.073

2021	Guadalmesí	1.094	0.095

2021	Guadalmesí	1.841	0.046

2021	Guadalmesí	1.190	0.054

2021	Guadalmesí	1.547	0.066

2024	Guadalmesí	1.286	0.062

2024	Guadalmesí	1.347	0.093

2024	Guadalmesí	1.485	0.074

2024	Guadalmesí	1.651	0.052

2024	Guadalmesí	1.416	0.064

2024	Guadalmesí	1.769	0.056

2024	Guadalmesí	1.169	0.058

2024	Guadalmesí	1.185	0.054

2024	Guadalmesí	1.060	0.101

2024	Guadalmesí	1.351	0.052

2024	Guadalmesí	1.173	0.081

2024	Guadalmesí	1.059	0.078

2024	Guadalmesí	1.722	0.061

2024	Guadalmesí	1.677	0.067

2024	Guadalmesí	1.573	0.080

2024	Guadalmesí	1.284	0.084

2024	Guadalmesí	1.069	0.076

2024	Guadalmesí	1.306	0.099

2024	Guadalmesí	1.543	0.066

2024	Guadalmesí	1.361	0.091

2011	Tarifa	1.281	0.063

2011	Tarifa	1.586	0.060

2011	Tarifa	1.263	0.088

2011	Tarifa	1.622	0.074

2011	Tarifa	1.307	0.099

2011	Tarifa	1.385	0.069

2011	Tarifa	1.855	0.045

2011	Tarifa	1.122	0.090

2011	Tarifa	1.487	0.074

2011	Tarifa	1.646	0.052

2011	Tarifa	1.248	0.107

2011	Tarifa	1.372	0.106

2011	Tarifa	1.351	0.074

2011	Tarifa	1.299	0.082

2011	Tarifa	1.882	0.041

2011	Tarifa	1.646	0.052

2011	Tarifa	1.195	0.098

2011	Tarifa	1.382	0.104

2011	Tarifa	1.799	0.031

2011	Tarifa	1.928	0.035

2014	Tarifa	1.391	0.068

2014	Tarifa	1.129	0.065

2014	Tarifa	1.495	0.090

2014	Tarifa	1.151	0.085

2014	Tarifa	1.486	0.074

2014	Tarifa	1.566	0.081

2014	Tarifa	1.187	0.054

2014	Tarifa	1.305	0.081

2014	Tarifa	1.574	0.080

2014	Tarifa	1.883	0.041

2014	Tarifa	1.002	0.089

2014	Tarifa	0.934	0.102

2014	Tarifa	1.186	0.099

2014	Tarifa	1.163	0.083

2014	Tarifa	1.851	0.045

2014	Tarifa	1.134	0.064

2014	Tarifa	1.259	0.088

2014	Tarifa	1.298	0.100

2014	Tarifa	1.608	0.035

2014	Tarifa	1.271	0.038

2018	Tarifa	1.220	0.074

2018	Tarifa	1.546	0.045

2018	Tarifa	1.524	0.069

2018	Tarifa	1.631	0.054

2018	Tarifa	1.479	0.092

2018	Tarifa	1.873	0.042

2018	Tarifa	1.589	0.060

2018	Tarifa	1.144	0.062

2018	Tarifa	1.117	0.067

2018	Tarifa	1.153	0.061

2018	Tarifa	1.422	0.099

2018	Tarifa	1.583	0.079

2018	Tarifa	1.408	0.066

2018	Tarifa	1.722	0.061

2018	Tarifa	1.493	0.053

2018	Tarifa	1.481	0.055

2018	Tarifa	1.551	0.083

2018	Tarifa	1.395	0.087

2018	Tarifa	1.694	0.046

2018	Tarifa	1.685	0.047

2021	Tarifa	1.250	0.068

2021	Tarifa	1.719	0.062

2021	Tarifa	1.419	0.100

2021	Tarifa	1.269	0.065

2021	Tarifa	1.233	0.092

2021	Tarifa	1.492	0.073

2021	Tarifa	1.153	0.061

2021	Tarifa	1.246	0.069

2021	Tarifa	1.284	0.084

2021	Tarifa	1.623	0.055

2021	Tarifa	1.005	0.089

2021	Tarifa	1.173	0.082

2021	Tarifa	1.106	0.069

2021	Tarifa	1.274	0.064

2021	Tarifa	1.326	0.056

2021	Tarifa	1.801	0.052

2021	Tarifa	1.298	0.100

2021	Tarifa	1.538	0.085

2021	Tarifa	1.233	0.045

2021	Tarifa	1.458	0.033

2024	Tarifa	1.477	0.076

2024	Tarifa	1.492	0.053

2024	Tarifa	1.278	0.103

2024	Tarifa	1.175	0.056

2024	Tarifa	1.063	0.078

2024	Tarifa	1.825	0.048

2024	Tarifa	1.220	0.074

2024	Tarifa	1.542	0.084

2024	Tarifa	1.540	0.085

2024	Tarifa	1.249	0.042

2024	Tarifa	1.289	0.101

2024	Tarifa	1.336	0.077

2024	Tarifa	1.127	0.066

2024	Tarifa	1.042	0.082

2024	Tarifa	1.527	0.069

2024	Tarifa	1.935	0.035

2024	Tarifa	1.432	0.098

2024	Tarifa	1.203	0.097

2024	Tarifa	1.086	0.114

2024	Tarifa	1.485	0.029
